# Associations between adiposity, diabetes, lifestyle factors and the risk of gliomas

**DOI:** 10.3389/fmed.2023.1207223

**Published:** 2023-07-11

**Authors:** Xiaozhi Liu, Yang Wang, Yuxiang Wang, Jincheng Zhao, Wanchao Shi, Yujun Zhao, Lei Chen, Lei Wu

**Affiliations:** ^1^Department of Neurosurgery, Tianjin Fifth Central Hospital, Tianjin, China; ^2^Tianjin Key Laboratory of Epigenetic for Organ Development of Preterm Infants, Tianjin Fifth Central Hospital, Tianjin, China; ^3^Department of Neurology, The First Hospital of Qinhuangdao, Qinhuangdao, China; ^4^Department of Neurosurgery, The First Hospital of Qinhuangdao, Qinhuangdao, China; ^5^Cerebrovascular Interventional Treatment Ward, Tianjin Fifth Central Hospital, Tianjin, China; ^6^Department of Neurocritical Medicine, Tianjin Fifth Central Hospital, Tianjin, China

**Keywords:** adiposity, diabetes, lifestyle factors, gliomas, Mendelian randomization

## Abstract

**Background:**

Despite numerous observational studies linking adiposity, diabetes, and lifestyle factors with gliomas, the causal associations between them remain uncertain.

**Methods:**

This study aimed to use two-sample Mendelian randomization (MR) analysis to investigate whether these associations are causal. Specifically, independent genetic variants in body mass index (BMI), waist circumference (WC), type 2 diabetes (T2D), smoking, alcohol, and coffee consumption were extracted from the published genome-wide association studies (GWASs) with genome-wide significance. The corresponding summary-level data for gliomas were available from a GWAS of 1,856 cases and 4,955 controls of European descent from the GliomaScan consortium. Additionally, glioma pathogenesis-related protein 1 data were used for validation, and Radial MR analysis was conducted to examine the potential outlier single-nucleotide polymorphisms (SNPs).

**Results:**

One standard deviation (SD) increase in BMI had an odds ratio (OR) of 1.392 (95% confidence interval (CI), 0.935–2.071) for gliomas, while one SD increase in WC had an OR of 0.967 (95% CI, 0.547–1.710). For T2D, a one-unit increase in log-transformed OR had an OR of 0.923 (95% CI, 0.754–1.129). The prevalence of smoking initiation had an OR of 1.703 (95% CI, 0.871–3.326) for gliomas, while the prevalence of alcohol intake frequency had an OR of 0.806 (95% CI, 0.361–1.083), and the prevalence of coffee intake had an OR of 0.268 (95% CI, 0.033–2.140) for gliomas.

**Conclusion:**

This study provides evidence that adiposity, T2D, smoking, alcohol drinking, and coffee intake do not play causal roles in the development of gliomas. The findings highlight the importance of reconsidering causal relationships in epidemiological research to better understand the risk factors and prevention strategies for gliomas.

## Introduction

Malignant tumors of the central nervous system (CNS) usually have the poorest prognosis among all cancers, leading to the highest estimated life lost when compared with any other cancers (1). Gliomas are the most common CNS tumors (2), accounting for about 80% of all malignant tumors and 30% of all primary brain ones. Besides, gliomas have the highest death rate of all deaths caused by primary brain tumors (3, 4). Several possibly adjustable factors for gliomas have been revealed in some epidemiological studies, including adiposity (5, 6), alcohol consumption (7), smoking (8, 9), diabetes (10, 11), and coffee intake (12). The role of insulin resistance, inflammation, and altered hormone levels may take part in the potential mechanisms, underlying the association between adiposity, diabetes, lifestyle factors, and glioma risk (5–12).

Previous research on the association between the abovementioned factors and the risks of gliomas has been inconsistent, with some studies reporting significant associations while others have found no association (8, 13). The inconsistency could be caused by a variety of factors. But we believe that the most significant one is that all of these studies are observational ones. Therefore, the observational conclusions could be influenced by some unknown confounders, misclassification, and even reverse causality (14). Determining the causal effect of potentially modifiable risk factors with gliomas is very important for us to understand the etiology of this disease, and also to help us prevent and manage the disease in clinical practice.

Mendelian randomization (MR) design is a powerful tool to extract genetic variants from previously published genome-wide association studies (GWASs) as instrumental variables (IVs) to determine the causal inference between exposures and outcomes (15). The MR study can reduce the residual confounding because the genetic IVs were randomly assorted at conception, which was finished before the onset of the diseases or other environmental factors (16). In addition, the MR study is a useful tool to minimize reverse causality since the germline variants are used in the MR analysis and the genetic IVs will not change during the development or progression of the disease (17).

In this study, a two-sample MR study was performed to examine the causal associations between adiposity, diabetes, smoking and alcohol, and coffee intake and the risk of gliomas.

## Methods

### Study design

**Figure 1:**
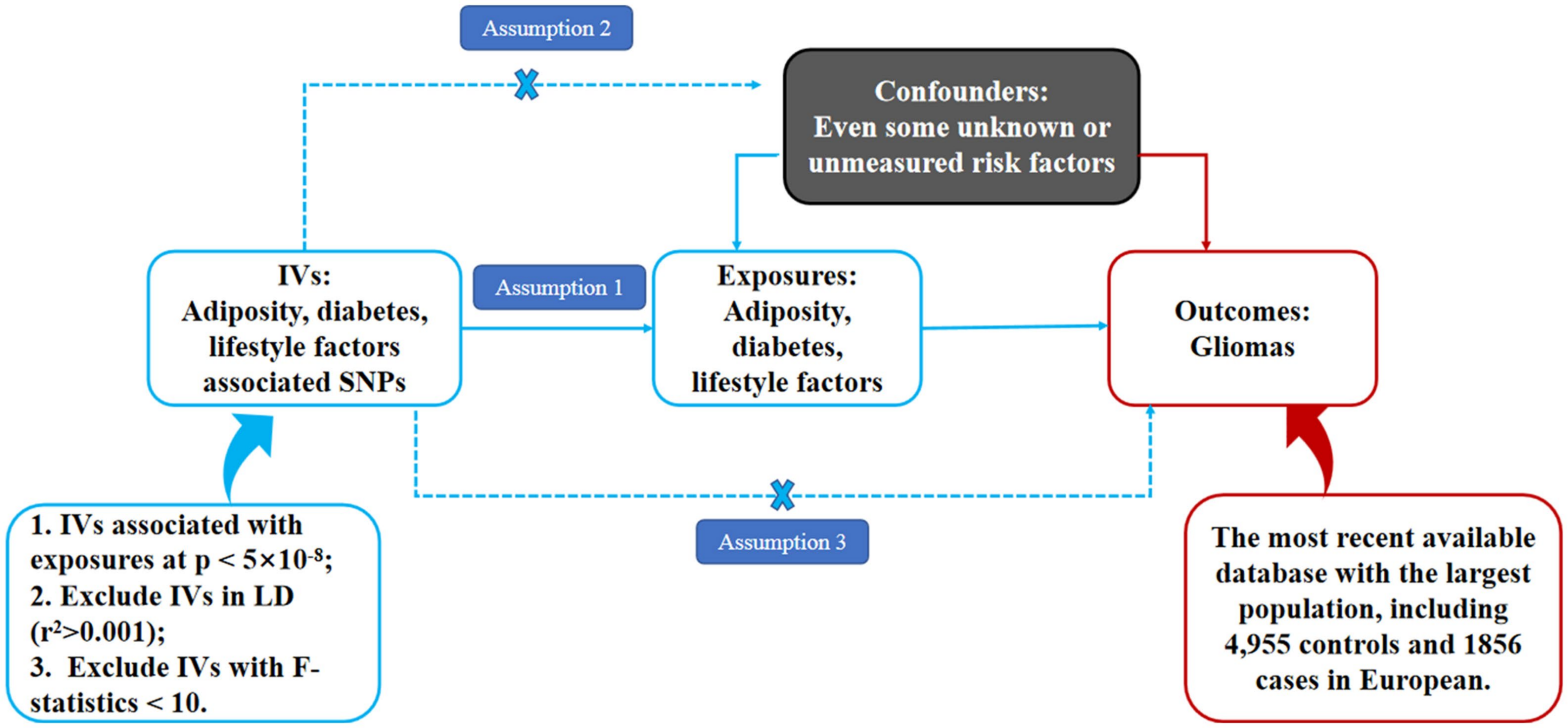
Schematic representation of an MR analysis. We selected independent SNPs associated with adiposity, diabetes, and lifestyle factors at a genomic significance of 5 × 10^−8^ from the published genome-wide association studies (GWASs), and the corresponding risks for gliomas were obtained from the GliomaScan consortium. The first assumption of MR analysis is that the genetic variants used as instrumental variables (IVs) are robustly and significantly associated with exposure (adiposity, diabetes, and lifestyle factors). The second assumption is that the used IVs are not significantly associated with any confounders. The third assumption is that the selected IVs can only affect the risk of the outcome (gliomas) merely through the exposures (adiposity, diabetes, and lifestyle factors), not via alternative pathways.

Independent IVs significantly associated with adiposity, diabetes, and lifestyle factors were enrolled in the present MR analysis. These IVs were delivered to the gametocyte randomly and independently at meiosis, which is similar to the design of randomized control trials. Therefore, MR can be used to detect the causal associations between exposures (adiposity, diabetes, and lifestyle factors) and outcomes (gliomas) based on the GWAS data in a retrospective way. In the present MR analysis, adiposity was valued through body mass index (BMI) and waist circumference (WC). As is shown in Figure 1, this study was an MR study based on summary-level data from previously published GWAS data of adiposity (including both overall and central adiposity), type 2 diabetes, lifestyle factors (including smoking and alcohol, and coffee intake), and gliomas. All the original studies included in the present MR had been approved by the Ethics Committee of their institutions, respectively. Besides, informed consent had been obtained from the involved participants in their original studies. Therefore, no further ethical approval is required in the present MR analysis (15–17).

### Instrument variable selection

The single nucleotide polymorphisms (SNPs), which were robustly and significantly (at a genome-wide significance level of *p* < 5 × 10^−8^) associated with BMI (18), WC (output from the GWAS pipeline using Phesant derived variables from UKBiobank), smoking initiation (19), alcohol drinking and coffee intake (both alcohol drinking and coffee intake are output from the GWAS pipeline using Phesant derived variables from UKBiobank), and type 2 diabetes (T2D) (20), were used as IVs and obtained from the largest and newest GWASs (Table 1). Smoking initiation was defined as a categorical variable, including regular cigarette smokers (current or past smokers) and individuals who did not smoke cigarettes regularly. In addition to assumption 1 (robust and significant IVs), the extract_instruments functions were used to find the GWAS-significant SNPs for a specified set of outcomes, which contains the LD-based clumping (with a threshold of *r*^2^ < 0.001 and clumping window >10,000 kb). Therefore, only independent significant associations were returned after the extract_instruments functions.

**Table 1:** Information on used studies and consortia.

Exposure or outcome	Unit	Participants	Identified SNPs	All SNPs
Body mass index (18)	SD	681,275	30	2,336,260
Waist circumference^a^	SD	462,166	249	9,851,867
Type 2 diabetes (20)	Log OR	61,714 cases and 1,178 controls	19	5,030,727
Smoking initiation (19)	Log OR	311,629 cases and 321,173 controls	36	607,291
Alcohol intake frequency^a^	SD	462,346	59	9,851,867
Coffee intake^a^	Log OR	428,860	129	9,851,867
Glioma (21)	Log OR	1,856 cases and 4,955 controls	—	309,636

SD, Standard deviation; OR, odds ratio. All of the studies were finished in the European populations and built on the HG19/GRCh37.

a The SNPs were extracted from the GWAS pipeline using Phesant-derived variables from UKBiobank.

### Gliomas data source

The summary-level data of exposure-related SNPs in gliomas (21) were obtained from a genome-wide association meta-analysis of the GliomaScan consortium, which included a total of 1,856 glioma cases and 4,955 controls of European descent (21). The GliomaScan consortium finished the meta-analysis by obtaining data from 3 case-control studies, 14 cohort studies, and 1 population-based case-only study. To minimize the potential bias to glioma with longer survival, the cohort studies with a large number of incident cases (556 out of 1856, i.e., 30% of all cases) were also included by the GliomaScan consortium included in the genome-wide association meta-analysis. Therefore, the 556 incident cases were also a part of the 1,856 cohort used in the study. The detailed information for enrolled glioma patients is just shown in Supplementary Table S1.

To verify the reliability of our results, we further used the data of glioma pathogenesis-related protein 1 (GLIPR1) for supplementary analysis to explore the causal association between adiposity, diabetes, lifestyle factors, and the level of glioma pathogenesis-related protein 1. The SNPs associated with glioma pathogenesis-related protein 1 were obtained from the genomic atlas of the human plasma proteome based on the INTERVAL study, which included 3,301 healthy blood donors.

### Statistical analysis

The MR analysis must satisfy the following three basic assumptions: assumption one: the IVs must be significantly associated with our interested exposures (such as adiposity, diabetes, and lifestyle factors in the present MR analysis); Assumption two: the IVs must not be associated with any other confounding factors in the pathway between exposure and outcome, and assumption three: the IVs affect our interested outcome (such as gliomas) entirely through our exposures.

In the present MR analysis, the inverse variance weighted method was used as the principal statistical analysis, which assumed that all the enrolled IVs were valid. However, in some situations, not all the IVs were valid. Therefore, sensitivity analyses were necessary for us to detect the stability and reliability of our analysis. The different MR analyses were based on different heterogeneity and horizontal pleiotropy. The weighted median method can provide an effective estimate based on >50% of weight from valid SNPs (22). The MR-Egger analysis can provide us with a pleiotropy-corrected estimate if the horizontal pleiotropy was significant (*p* for intercept <0.05). Therefore, both the weighted median and MR-Egger were used in the sensitivity analyses. However, the MR-Egger may be underpowered in such conditions (22). Therefore, the consistency of these analyses suggests the stability and reliability of the present MR. Moreover, the mr_heterogeneity was used to test the heterogeneity and the mr_pleiotropy_test was used to detect the horizontal pleiotropy of this MR analysis. Besides, the mRnd was used to calculate the power to detect the power of the present MR analysis, which is available at https://shiny.cnsgenomics.com/mRnd/.

## Results

### Genetic instrumental variables for exposures

As shown in Supplementary Table S2, all genetic IVs associated with BMI (*N* = 249), WC (*N* = 129), T2D (*N* = 59), smoking (*N* = 36), alcohol drinking (*N* = 30), and coffee intake (*N* = 19) are presented. All of these IVs were significantly associated with our exposures with *F* statistics >10.

### Mendelian randomization analyses for gliomas

**Figure 2:**
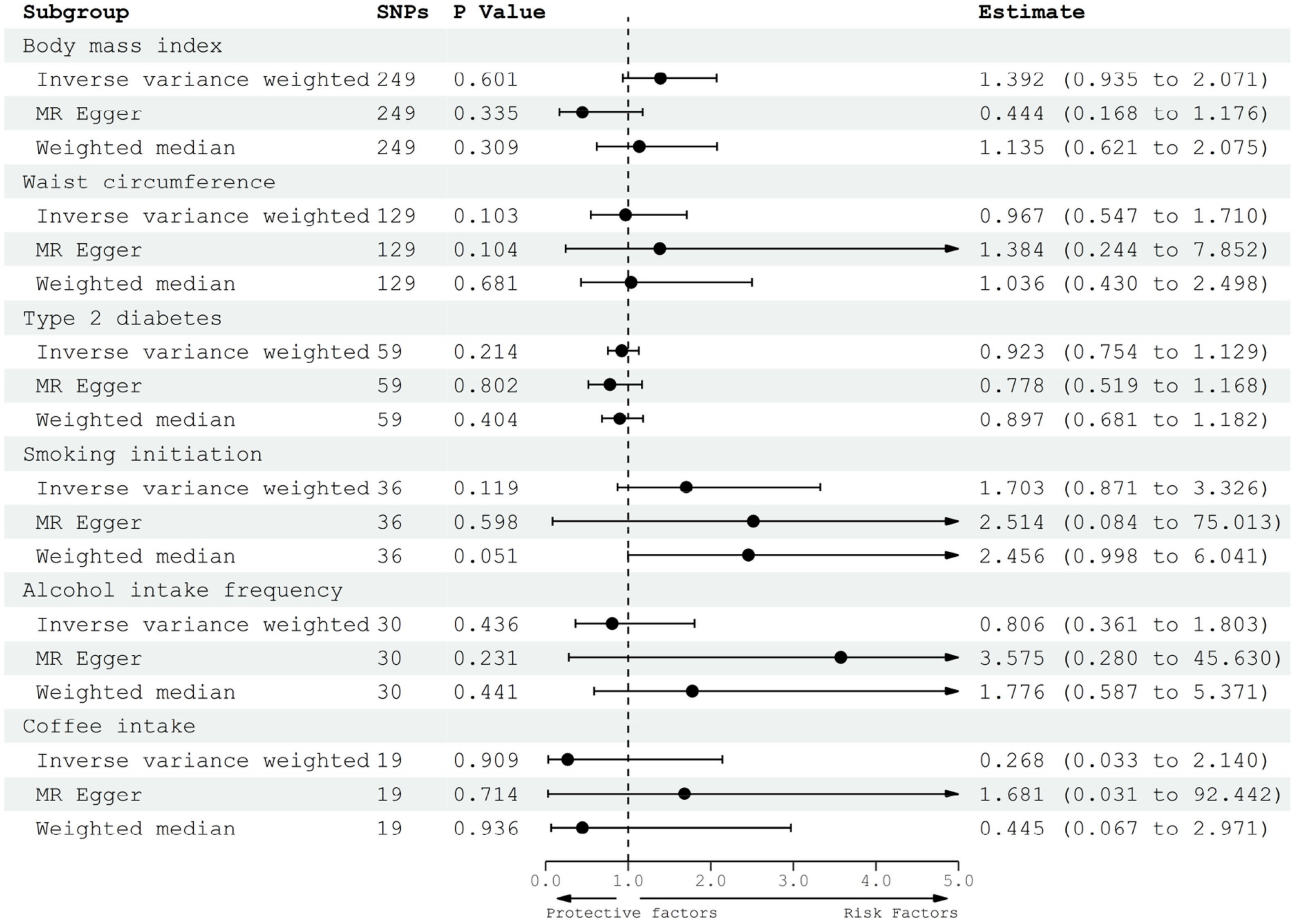
Forest plot to visualize the causal effect of adiposity, diabetes, and lifestyle factors on the risk of gliomas.

In the principle analysis, our results revealed that the odds ratio (OR) of gliomas was 1.392 [95% confidence interval (CI), 0.935–2.071] for one standard deviation (SD) increase in BMI, 0.967 (95% CI, 0.547–1.710) for one SD increase in WC, 0.923 (95% CI, 0.754–1.129) for a one-unit increase in log-transformed OR of T2D, 1.703 (95% CI, 0.871–3.326) for one SD increase in the prevalence of smoking initiation, 0.806 (95% CI, 0.361–1.083) for one SD increase in the prevalence of alcohol intake frequency, and 0.268 (95% CI, 0.033–2.140) for one SD increase in the prevalence of coffee intake (Figure 2). Besides, the power of all the analyses (except the WC and T2D) was higher than 0.8 in mRnd analyses, suggesting that the sample sizes were adequate for detecting meaningful associations. However, the causal effects of WC and T2D on gliomas need further validation in larger populations.

### Sensitive analysis, heterogeneity, and horizontal pleiotropy analysis

MR-Egger and weighted median regression were performed for the sensitive analysis (Figure 2). All three MR methods showed that the genetically predicted adiposity, diabetes, and lifestyle factors have no significant causal effect on the risk of gliomas. Therefore, all three MR methods achieved consistent conclusions even based on different levels of valid SNPs. Besides, the mr_pleiotropy test was performed to verify the pleiotropy, which meant that we could find whether these IVs were associated with other confounding factors. By the mr_pleiotropy test, there was no significant directional pleiotropy found in all of the MR-Egger intercepts (*p* > 0.05), suggesting no directional pleiotropic effects existed in the MR analysis.

### Effects of individual genetic instruments

In the leave-one-out analysis, every independent and significant SNP will be removed one by one, and then the inverse-variance weighted (IVW) analysis will be repeated on the remaining SNPs, which can be used to determine the effect of every single SNP on the total estimate. In the present leave-one-out analysis, no significant difference was detected. Therefore, our results were robust and reliable, which would not be influenced by any single genetic SNP.

### Validation in glioma pathogenesis-related protein 1

To finish the validatory statistical analysis using different independent cohorts, GLIPR1 was considered as another outcome. The results showed that the correlation coefficient (*β* value) between the glioma pathogenesis-related protein 1 and BMI were 0.045 (*p* = 0.562), and − 0.046 (*p* = 0.627) for WC, −0.019 (*p* = 0.594) for T2D, −0.009 (*p* = 0.935) for smoking initiation, 0.123 (*p* = 0.683) for alcohol intake frequency, and − 0.174 (*p* = 0.539) for coffee intake (Table 2) with no obvious heterogeneity and horizontal pleiotropy.

**Table 2:** The causal effect of adiposity, diabetes, and lifestyle factors on the levels of glioma pathogenesis-related protein 1.

Exposures	MR methods	Nsnp	*b*	se	*p*
Type 2 diabetes	MR Egger	118	0.004	0.082	0.964
Type 2 diabetes	Weighted median	118	0.021	0.069	0.761
Type 2 diabetes	Inverse variance weighted	118	−0.019	0.036	0.594
Body mass index	MR Egger	505	−0.022	0.205	0.915
Body mass index	Weighted median	505	−0.057	0.128	0.658
Body mass index	Inverse variance weighted	505	0.045	0.077	0.562
Smoking initiation	MR Egger	91	0.463	0.576	0.423
Smoking initiation	Weighted median	91	−0.052	0.166	0.754
Smoking initiation	Inverse variance weighted	91	−0.009	0.112	0.935
Coffee intake	MR Egger	40	−0.245	0.572	0.670
Coffee intake	Weighted median	40	−0.344	0.402	0.392
Coffee intake	Inverse variance weighted	40	−0.174	0.284	0.539
Alcohol intake frequency	MR Egger	98	0.066	0.269	0.808
Alcohol intake frequency	Weighted median	98	0.004	0.215	0.985
Alcohol intake frequency	Inverse variance weighted	98	−0.050	0.123	0.683
Waist circumference	MR Egger	370	−0.095	0.271	0.727
Waist circumference	Weighted median	370	−0.167	0.172	0.332
Waist circumference	Inverse variance weighted	370	−0.046	0.095	0.627

Nsnp, the numbers of single-nucleotide polymorphisms.

### Radial MR analysis to examine potential outlier SNPs

**Figure 3:**
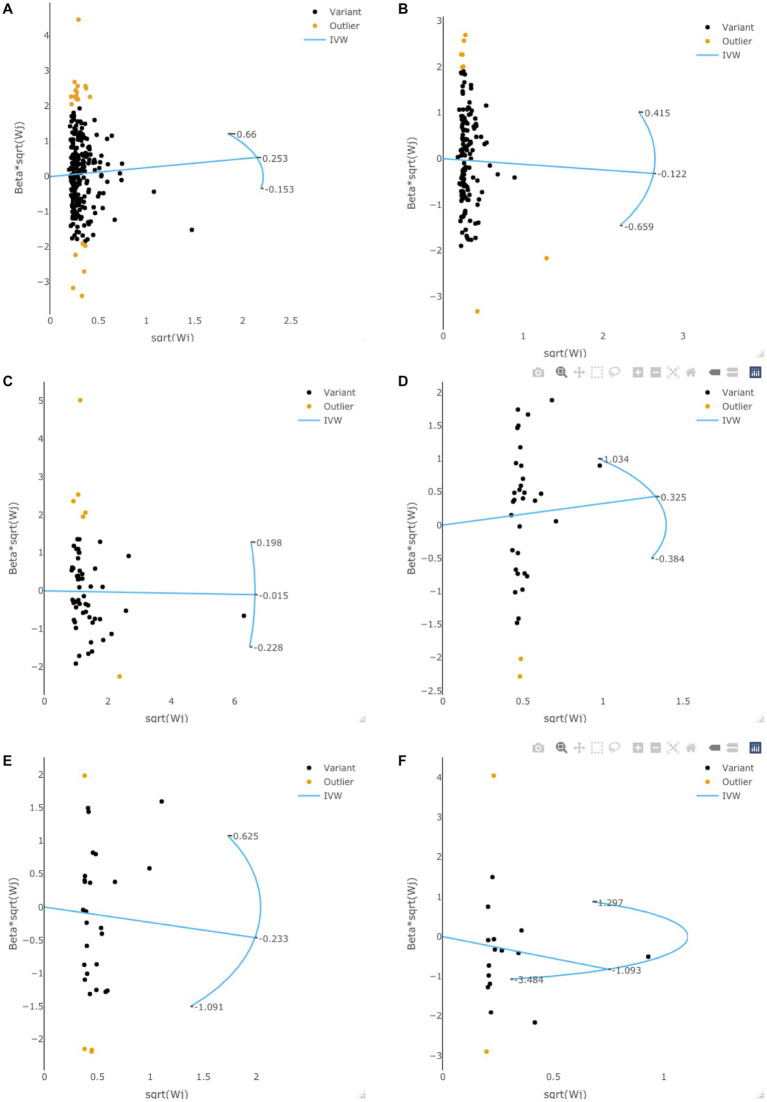
The radial MR plots in exploring potential outlier SNPs and their effects on gliomas. This figure showed the radial MR plots in body mass index **(A)**, waist circumference **(B)**, type 2 diabetes **(C)**, smoking initiation **(D)**, alcohol intake frequency **(E)**, and coffee intake **(F)**.

Radial MR analysis was performed to examine the potential outlier SNPs. As is shown in Figure 3, some potential outlier SNPs may affect our results in exploring the causal effects of adiposity, diabetes, and lifestyle factors on the risk of gliomas. However, the *F*-statistic (all *p* > 0.05) and *Q*-statistic (all *p* > 0.05) showed that the total effect size is reliable and not heterogeneous. Besides, all of the sensitivity analyses achieved consistent conclusions, suggesting that these outlier SNPs would not change our conclusions.

## Discussion

This is the first study to explore the causal associations between genetically predicted BMI, WC, T2D, smoking, alcohol drinking, coffee intake, and the risk of gliomas using MR design. And our results showed that there were no causal roles of adiposity, diabetes, lifestyle factors, and the risk of gliomas.

Although many observational studies have explored the associations between the specific diet or certain other lifestyle choices and the risk of the development of glioma (3), little consensus has been achieved since the inconsistency of these conclusions in different studies.

Many studies have been designed to evaluate the potential association between adiposity and the risk of glioma (including general and abdominal adiposity). A nationwide population-based cohort study of Koreans enrolled 6,833,744 people older than 20 years and 4,771 glioma cases were documented during the median follow-up period of 7.30 years (5), which revealed that individuals with higher BMI and WC had a higher risk of glioma. Besides, a meta-analysis enrolled 22 studies (including 2,418 glioma cases in a total cohort size of 10,143,803 subjects) and 8 case-control studies (1,265 glioma cases and 8,316 controls) (6). This study found that adiposity (measured by BMI) was a risk factor for gliomas among females and no such associations in males (6). The authors concluded that controlling adiposity may help reduce the risk of developing glioma (5, 6). Observational studies on the associations between lifestyle factors and glioma risk have yielded inconsistent results, which can be attributed to several factors. Firstly, observational studies are prone to bias, including selection bias, recall bias, and confounding. Selection bias occurs when the study population is not representative of the general population, leading to biased estimates of the association between the exposure and outcome variables. Recall bias occurs when participants have difficulty recalling past exposures accurately, leading to misclassification of exposure status. Confounding occurs when there are other factors that are associated with both the exposure and outcome variables, leading to spurious associations between the two variables. Secondly, observational studies often have small sample sizes, which may limit their statistical power to detect a true association between the exposure and outcome variables. This can lead to inconsistent results across studies. Thirdly, observational studies often rely on self-reported data, which may be subject to social desirability bias. Participants may provide responses that they believe are socially acceptable, rather than their true behaviors or exposures.

MR analysis can help address some of these limitations of observational studies. By using genetic variants as instrumental variables, MR analysis can minimize the impact of confounding and reverse causation, as genetic variants are randomly assigned at conception and are not affected by environmental or lifestyle factors. Our MR revealed that the genetically predicted BMI and WC were not causally associated with a higher risk of gliomas.

Glioma, a rare brain tumor originating from glial cells, affects a small proportion of the population. It is a relatively rare condition, accounting for only about 1% of all cancers and affecting approximately 5 in 100,000 people per year (10). In contrast, diabetes, a metabolic disease, is a known risk factor for many types of malignant tumors (23, 24), including gliomas (10), affecting around 463 million adults worldwide (23, 24). Interestingly, recent research has revealed a potential association between glioma and diabetes. Several studies have investigated the association between diabetes and glioma risk, and some have also examined the relationship between diabetes and glioma survival (25). However, the findings from these studies are contradictory. While some studies suggest that long-term diabetes may reduce the risk of glioma (26), others found no association between diabetes and glioma survival (27). In our MR analysis, we found no causal relationship between genetically predicted diabetes and the development of gliomas. However, the causal relationship between diabetes and glioma survival was not assessed in our study.

Except for adiposity and diabetes, other potential lifestyle factors (smoking, alcohol drinking, and coffee intake) have also been investigated, but no consistent associations were found in the previous studies (3, 4, 9, 13). In the present MR analysis, we also found no causal associations between lifestyle factors and the risk of gliomas. With regard to smoking, the gender-specific difference also existed. Li et al. (8) revealed an increased risk of gliomas in past female smokers but not in males. However, it is a pity that we cannot reconfirm such gender-specific differences in our MR analysis.

GLIPR1 is a tumor suppressor protein that has been implicated in the development and progression of glioma. Studies have shown that GLIPR1 expression is reduced in glioma cells compared to normal glial cells, and that restoration of GLIPR1 expression can inhibit glioma cell growth and induce apoptosis (28). Additionally, GLIPR1 has been shown to regulate various signaling pathways involved in glioma development and progression, including the PI3K/Akt and MAPK/ERK pathways (28). Therefore, GLIPR1 was considered as a promising target for the development of new therapies for glioma treatment. In the present study, the levels of GLIPR1was used to validate the causal effect of adiposity, diabetes, and lifestyle factors on the risk of gliomas. Similar to the findings in the main analysis, no causal association was observed, which further confirmed that there was no causal relationship between them.

In addition, glioma is characterized by numerous tumor-driver genes and distinct pathological subtypes. Previous cancer genomics studies, such as the GliomaScan study, have revealed compelling evidence supporting the genetic etiology of gliomas, including loci such as 20q13.33 (RTEL), 5p15.33 (TERT), and 9p21.3 (CDKN2BAS), as well as both loci of 7p11.2 (EGFR), 8q24.21 (CCDC26), and 11q23.3 (PHLDB1) (29). Nevertheless, we conducted a thorough search for all included SNPs in the exposures to ensure that none of them was significantly associated with glioma. This crucial assumption was necessary to ensure the reliability and robustness of our findings in the MR analysis. As a result, our study provides reliable and robust results, even in the radial MR analysis.

There are several strengths in the present MR analysis: first, only independent SNPs significantly associated with the exposures (including adiposity, diabetes, and lifestyle factors) were used in our MR analysis to satisfy the basic assumptions of MR. Besides, all the GWASs of exposures and outcomes were finished just in European ancestry populations to reduce the potential confounder. Most importantly, MR Egger regression was performed and no evidence of directional pleiotropic effects was found. Therefore, the results of the present MR are valid and robust.

Several potential limitations existed in the present MR. First, only summary-level statistics rather than individual-level data were used in this MR. Therefore, we cannot further explore the gender-specific association between adiposity, diabetes, lifestyle factors, and the risk of gliomas. Besides, this study was finished based on the data of European ancestry populations. Whether these associations existed in other ancestry populations needs further investigation due to the genetic variance in different ancestry populations (30). The potential for bias may also limit the generalizability of our findings to other populations. Future research should be conducted to address these limitations. We also tried to replicate our analysis in the Chinese Glioma Genome Atlas (CGGA) dataset (31). However, the CGGA dataset only contains whole-exome sequencing data in 286 patients and the power is too low to provide a convincing conclusion. Therefore, if more cases finished the whole-exome sequencing in the CGGA database, it may provide us with more evidence by replicating the MR analysis in this database. In the validation of the causal associations with the levels of glioma pathogenesis-related protein 1, causality was still not observed, which was consistent with the findings in glioma.

## Conclusion

Our results suggest that there are no causal effects of adiposity, T2D, smoking, alcohol drinking, and coffee intake on the development of gliomas. Therefore, strategies aimed at reducing the risk of gliomas through the modification of these factors may not be effective, and alternative approaches should be explored.

## Data availability statement

The original contributions presented in the study are included in the article/Supplementary material, further inquiries can be directed to the corresponding author.

## Ethics statement

Ethical review and approval was not required for the study on human participants in accordance with the local legislation and institutional requirements. Written informed consent from the (patients/ participants OR patients/participants legal guardian/next of kin) was not required to participate in this study in accordance with the national legislation and the institutional requirements.

## Author contributions

LW designed the study and revised the manuscript. XL and YW finished the analysis. YW and JZ helped with the use of the software. WS, YZ, and LC finished the draft of this manuscript. All authors contributed to the article and approved the submitted version.

## Funding

This study was supported by grant from Tianjin Science and Technology Plan Project (Grant No. 22ZYQYSY00030), Tianjin Natural Science Foundation (Grant No. 21JCZDJC01270), Tianjin Health Technology Project (Grant No. TJWJ2022XK043), Bethune Charity Foundation Project (Grant No. B-0307-H20200302), and Tianjin Key Medical Discipline (Specialty) Construction Project (TJYXZDXK-062B).

## Acknowledgments

We acknowledged Union_of_Researchers (WeChat Subscription) for his help with the methods of our analysis.

## Conflict of interest

The authors declare that the research was conducted in the absence of any commercial or financial relationships that could be construed as a potential conflict of interest.

## Publisher’s note

All claims expressed in this article are solely those of the authors and do not necessarily represent those of their affiliated organizations, or those of the publisher, the editors and the reviewers. Any product that may be evaluated in this article, or claim that may be made by its manufacturer, is not guaranteed or endorsed by the publisher.
